# Real-Time Vision-Based Fall Detection Systems for the Elderly: A Systematic Review

**DOI:** 10.3390/s26165069

**Published:** 2026-08-10

**Authors:** Mahammad Nabizade, Réda Yahiaoui, Isabelle Lajoie, Nassima Nacer, Frédéric Auber, Moustafa Fayad

**Affiliations:** 1 SINERGIES (UR 4662), Université Marie et Louis Pasteur, 25030 Besançon, France; reda.yahiaoui@univ-fcomte.fr (R.Y.); isabelle.lajoie@univ-fcomte.fr (I.L.); moustafa.fayad@univ-fcomte.fr (M.F.); 2 LyRIDS, ECE, 75015 Paris, France; fatma-zohra.nacer@ece.fr; 3 Department of Pediatric Surgery, SINERGIES (UR 4662), Université Marie et Louis Pasteur, CHU Besançon, 25030 Besançon, France; frederic.auber@univ-fcomte.fr

**Keywords:** PRISMA, fall detection, computer vision, deep learning, real-time, edge devices

## Abstract

Falls represent a threat to older adults, overload healthcare systems, and reduce quality of life. Vision-based fall detection has advanced recently through deep learning, yet most proposed models lack validation on physical hardware and do not report inference-time metrics. This systematic review, following PRISMA and Kitchenham guidelines, targets this gap. We focus exclusively on vision-based systems that report inference speed on a specified device. We define real-time performance using a threshold of 10 fps, based on the reported duration of the critical fall phase in real-life falls. From 588 records across IEEE Xplore, ACM Digital Library, Web of Science Core Collection, and PubMed (2019–2024), only 11 met all inclusion criteria, highlighting how few studies validate real-time performance on physical hardware. The findings show that CNN-based architectures dominate algorithm choice, edge devices dominate deployment platforms, and optimization remains central to real-time inference on constrained hardware. Across these studies, we identify two persistent limitations: no real-world testing with older adults and reliance on small, controlled datasets with simulated falls.

## 1. Introduction

**Figure 1:**
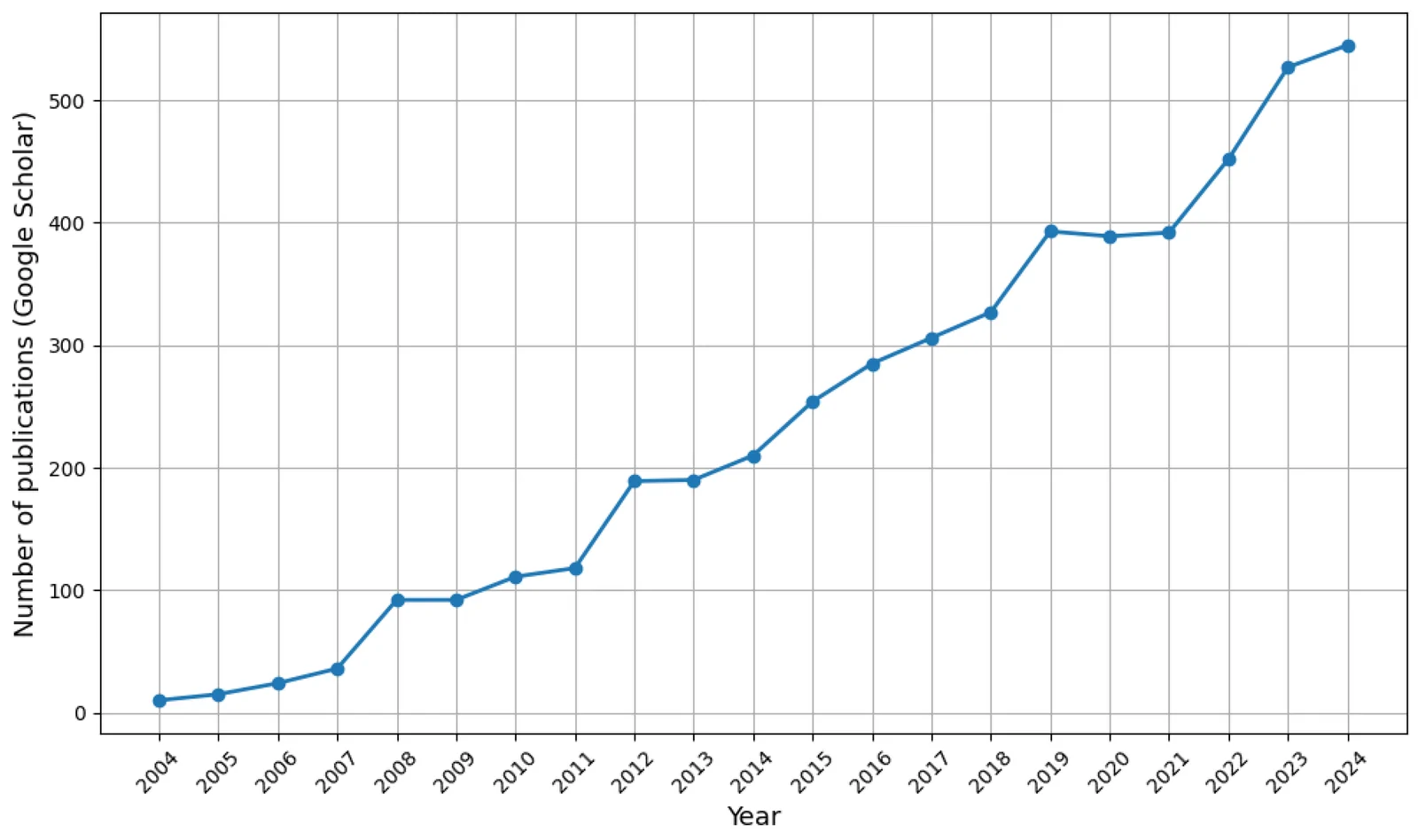
Yearly publications on fall detection indexed in Google Scholar (2004–2024). We queried allintitle:"fall detection" for each year separately and plotted the returned result counts without further filtering. Search performed on 20 April 2026.

The proportion of people aged 65 and older nearly doubled between 1974 and 2024 and will double again by 2074 [1]. Although longer life expectancy reflects social progress, it also increases demands on healthcare systems, which has driven a steady growth in elderly health research over the past two decades [2]. Fall detection has been a particularly active area within this trend. Figure 1 shows the general publication trend for fall detection, with the number of indexed titles growing from fewer than 20 in 2004 to more than 500 in 2024.

Over 30% of falls in older adults cause serious injuries, such as hip fractures or brain concussions, and many result in death [3]. In 2017, 8.4 million older adults in Western Europe required medical care due to fall-related injuries [4]. Effective fall detection systems could reduce these injury rates and ease pressure on healthcare systems.

Mubashir et al. [5] group fall detection systems into three categories: wearable, ambient, and vision-based. Wearable systems use accelerometers and gyroscopes in devices such as wristbands or pendants to track body movement [6]. However, wearable devices have limited battery life, require regular maintenance, and many older adults forget to wear them. Ambient systems rely on environmental sensors such as acoustic, passive infrared, and Doppler radar [7], but require costly installation and depend on specific environmental setups. Vision-based systems use cameras, including RGB, infrared, 3D, and depth sensors, to identify falls. They avoid the compliance and maintenance issues of wearable devices and the installation constraints of ambient systems. However, cameras raise privacy concerns, particularly in bedrooms and bathrooms, and system performance depends on camera placement, lighting, and occlusion conditions.

Vision-based fall detection increasingly relies on deep learning models such as convolutional neural networks (CNNs) and long short-term memory (LSTM) networks, which analyze visual representations including silhouettes, bounding boxes, and skeletal keypoints [8]. These models extract representations directly from raw data, removing the need for manual feature engineering and improving adaptability across scenes and subjects.

Because falls can cause serious injury or death, fall detection systems must operate with low latency. Yet existing models are predominantly evaluated in offline settings without real-time deployment or online data transmission [9], often in controlled environments that do not address practical challenges such as lighting variation or camera placement. This review addresses that gap: we systematically examine vision-based fall detection systems that demonstrate real-time inference on physical hardware and report measurable deployment metrics such as frames per second (fps).

This article is structured as follows: First, we summarize previous review articles. Next, we present the review questions and outline the methodology, including data sources, search strategy, and selection criteria. We then describe the quality assessment process. The results section addresses each research question, examining the algorithms used and the stages of deployment. Finally, we discuss the key findings and outline directions for future work.

## 2. Related Work

This section summarizes recent review articles on fall detection and identifies the gaps our review addresses.

Islam et al. [10] categorized deep learning-based fall detection systems into three main groups: CNN-based (e.g., 1D, 3D and optical flow variants), LSTM-based (e.g., LSTM with RNN or 3D CNN), and autoencoder-based systems. They summarized the datasets used across these studies and highlighted the challenges of deep learning-based fall detection. Although they discussed real-time usage, their analysis remained at a general level and did not evaluate device-specific constraints or inference-time metrics.

Wang et al. [9] surveyed the full data pipeline of fall detection systems, covering acquisition, transmission, fusion, and analysis. A key finding was the disparity in real-time capabilities: among 22 vision-based studies, only one demonstrated real-time alarm transmission, despite 40.9% claiming real-time performance without empirical evidence.

Gutiérrez et al. [2] examined vision-based fall detection systems published between 2015 and 2020. They analyzed system architectures, input signals, and classification methods. They also highlighted challenges such as dataset fragmentation and the gap between research and real-world applications.

Alam et al. [11] reviewed deep learning-based fall detection for assistive living, categorizing systems by model type: CNNs (including hybrid and autoencoder variants), LSTMs, and multilayer perceptrons. They examined implementation details, evaluation metrics, and optimization parameters across each category. They identified key limitations, including poor handling of multi-person and occlusion scenarios, limited consideration of privacy, and weak security measures, and recommended edge computing to enhance security. However, they did not analyze specific edge devices or the optimization trade-offs required for real-time inference on such hardware.

Gaya-Morey et al. [12] conducted a systematic review covering both fall detection and human activity recognition for elderly populations. The review addressed research questions related to deep learning and real-world deployment, including hardware requirements and privacy concerns. Their findings revealed that 50% of studies lacked privacy protection measures, and they emphasized the importance of model selection based on deployment conditions and dataset availability for reproducibility. While they addressed deployment-related aspects, their focus was primarily on input hardware and privacy rather than inference performance or processing optimization.

Table 1 summarizes these reviews. Although these studies have discussed various aspects of vision-based fall detection, only Gaya-Morey et al. [12] adopted a systematic review approach, and their scope included both fall detection and human activity recognition. Islam et al. [10] discussed real-time usage in general terms but did not evaluate device-specific constraints or inference-time metrics. Our review differs in that it focuses exclusively on vision-based fall detection with validated real-time performance, examining not only the algorithms used but also the deployment hardware, optimization strategies, and the trade-offs they introduce. Vision-based, as used in this review, refers to any system whose primary input is spatially resolved image or video data from one or more cameras, including RGB, RGB-D, thermal and event cameras. This excludes ambient sensors such as passive infrared detectors, which register presence or motion without producing image data.

## 3. Methods

For this review, we adopted the Kitchenham method for conducting systematic literature reviews [13] and reported our findings following the PRISMA 2020 guidelines [14]. We did not register this review, and we did not publish a separate review protocol. We defined the research questions, search strategy, and inclusion and exclusion criteria in advance and report them in full in this section.

### 3.1. Research Questions

We structured our systematic review around the following research questions:

- **RQ1:** Which algorithms are most commonly implemented in real-time vision-based fall detection systems?
- **RQ2:** What deployment platforms are used for vision-based fall detection systems and how do these choices influence real-time performance?
- **RQ3:** What optimization strategies are applied to achieve real-time inference in vision-based fall detection systems, and what trade-offs do they introduce?

RQ1 addresses the diversity of algorithmic approaches applied in this domain, identifying which model families appear most frequently in systems that achieve real-time inference.

RQ2 explores the relationship between deployment hardware and real-time performance. By examining deployment platforms, we aim to understand how hardware choices affect both inference speed and practical usability.

Finally, RQ3 examines the optimization strategies used to achieve real-time inference on resource-constrained devices. These strategies often involve trade-offs between speed, accuracy, and memory usage, and understanding them is important for practical deployment.

### 3.2. Data Sources

We selected four databases to cover technical and healthcare perspectives. We used IEEE Xplore and the ACM Digital Library for their strong coverage of computer science research, PubMed for healthcare-related studies, and Web of Science for its interdisciplinary scope.

### 3.3. Search Strategy

We chose keywords by pairing general phrases (“fall detection”, “fall accident”) with technical terms (“deep learning”, “CNN”, “machine learning”) and deployment-related keywords (“real-time”, “deployment”). To focus on vision-based approaches, we excluded articles whose titles contained ‘radar’, ‘accelerometer’, ‘gyroscope’, ‘wearable’, or ‘WiFi’ using NOT operators, and we excluded review articles to concentrate on original research contributions. We adapted the search strings to the query format of each database. Table 2 shows the query structure independent of database-specific syntax. It is important to note that our searches were restricted to the title field only. While this approach helped to focus the review on the most directly relevant studies, it may have led to the exclusion of some articles where “fall detection” or related deployment terms appeared only in the abstract or keywords.

**Figure 2:**
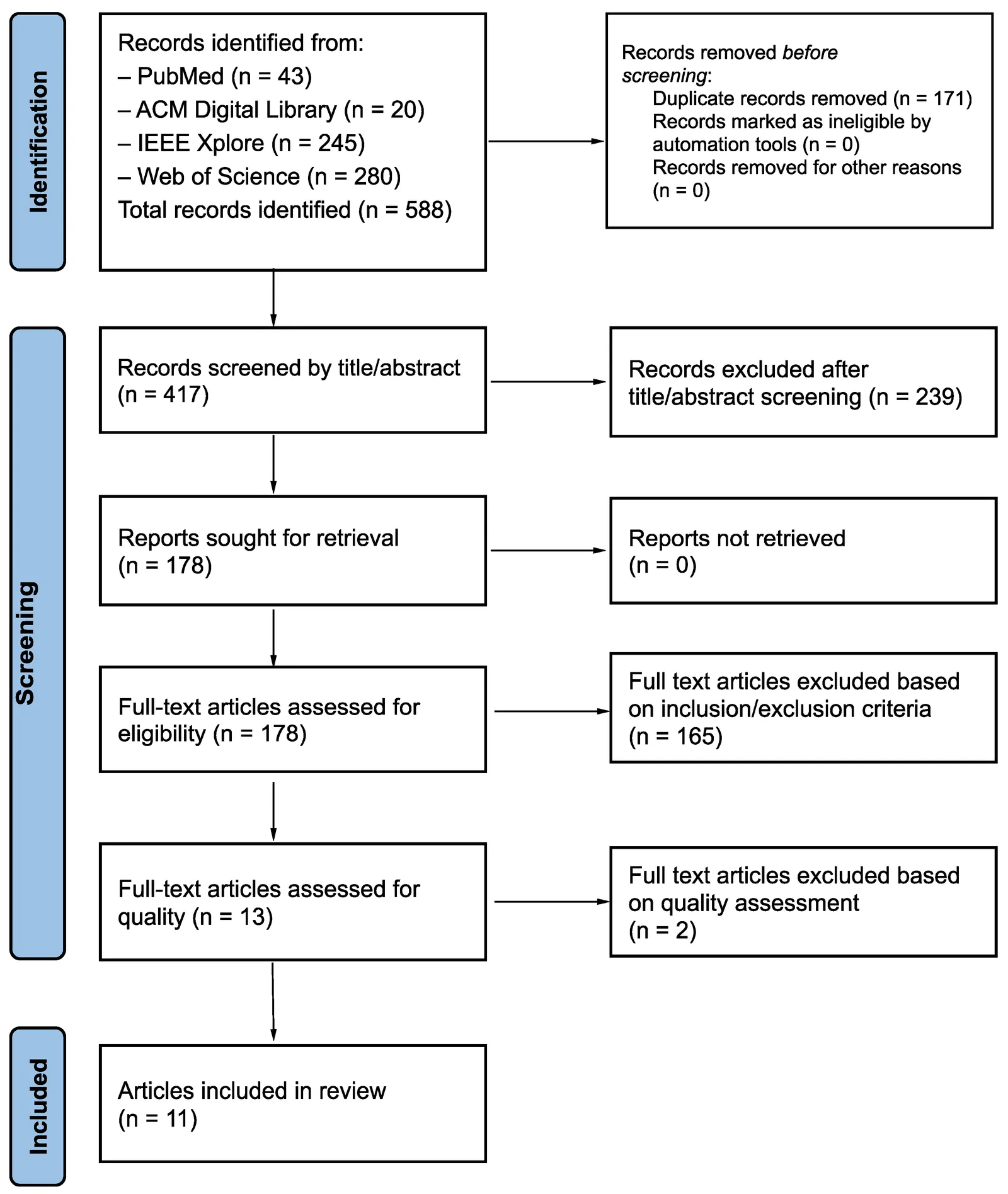
PRISMA flow chart for the study selection process.

The final search returned 588 records in total: 20 from the ACM Digital Library, 245 from IEEE Xplore, 43 from PubMed, and 280 from Web of Science. Figure 2 shows the PRISMA flow diagram for the study selection process.

### 3.4. Study Selection

Two authors (MF and MN) independently screened all records according to the predefined inclusion and exclusion criteria. Any disagreements were resolved through discussion between the two reviewers until consensus was reached.

- **Language**: We included only studies published in English.
- **Method**: We kept only vision-based work that used machine learning or deep learning, excluding fusion systems that combined vision with other modalities such as wearable sensors or ambient devices.
- **Target Population**:We included only studies that target elderly fall detection and excluded studies targeting other demographics such as construction workers or athletes. This criterion refers to the intended application, not the dataset composition; as Section 5 notes, none of the included datasets contain older-adult participants, and all falls were simulated by young volunteers.
- **Objective**: We excluded studies focused on human activity recognition or fall risk assessment to focus solely on fall detection systems.
- **Real-Time Capability**: We included studies reporting inference speeds of at least 10 fps on a specified test device. We derived this threshold from the reported timing of real-life falls. Vlaeyen et al. [15], who analyzed 26 real-life falls of older adults on video, found that the critical fall phase lasted on average 1946 ms (roughly 2 s). At 10 fps, a system processes approximately 20 frames during this phase, providing enough temporal resolution to capture the postural transition characteristic of a fall. This threshold does not guarantee clinical reliability, but it gives a clear lower bound for real-time screening. This value is consistent with prior deployed systems: Shu and Shu [16] reported that a minimum of 10 fps was required for accurate fall detection in their multi-camera system. Stone and Skubic [17] operated their fall detection system at 10 fps on dual-core Intel Atom CPUs in independent living facilities. We excluded studies that did not report fps or fell below this threshold.

During abstract screening, it was often unclear whether a study focused on deployment or whether other inclusion criteria were met. In such cases, we retained the record for full-text review and reapplied all criteria at that stage.

### 3.5. Quality Assessment

After the selection process, we evaluated the quality of each study to identify potential biases and confirm relevance to our research questions. Quality assessment was conducted independently by two authors (M.N. and M.F.), with disagreements resolved through discussion. Our quality assessment criteria include the following:

- **Implementation Details:** The study should describe the evaluation process in sufficient detail to assess the reported deployment results.
- **Comparative Evaluation:** The study should compare its performance with different network configurations (e.g., through ablation studies) or against other existing methods.
- **Evaluation Transparency:** The study should address potential biases such as dataset overfitting or evaluation limitations.

After applying these criteria, we assigned each study a score: 1 for ‘yes’, 0.5 for ‘partially’, and 0 for ‘no’. We retained articles with a total score of at least 2. The item-level quality assessment scores for all evaluated studies are provided in the Supplementary Material.

## 4. Results

After removing duplicates, applying the inclusion criteria, and conducting the quality assessment, we identified 11 studies from the initial 588 articles. Table 3 presents an overview of the selected studies.

### 4.1. RQ1: Algorithms

CNN-based models appear in all 11 studies, and we group them into 3 categories: CNN classifiers [18,28,31], CNN object detectors [24,25,32,33], and hybrid pipelines. Hybrid approaches combine CNN with optical flow [20] or with LSTM [22,27], whereas another study [29] uses a CNN-based pose estimator with machine learning methods. Across all three categories, the authors consistently prioritize low parameter counts and lightweight backbones to meet the constraints of their target devices.

**Figure 3:**
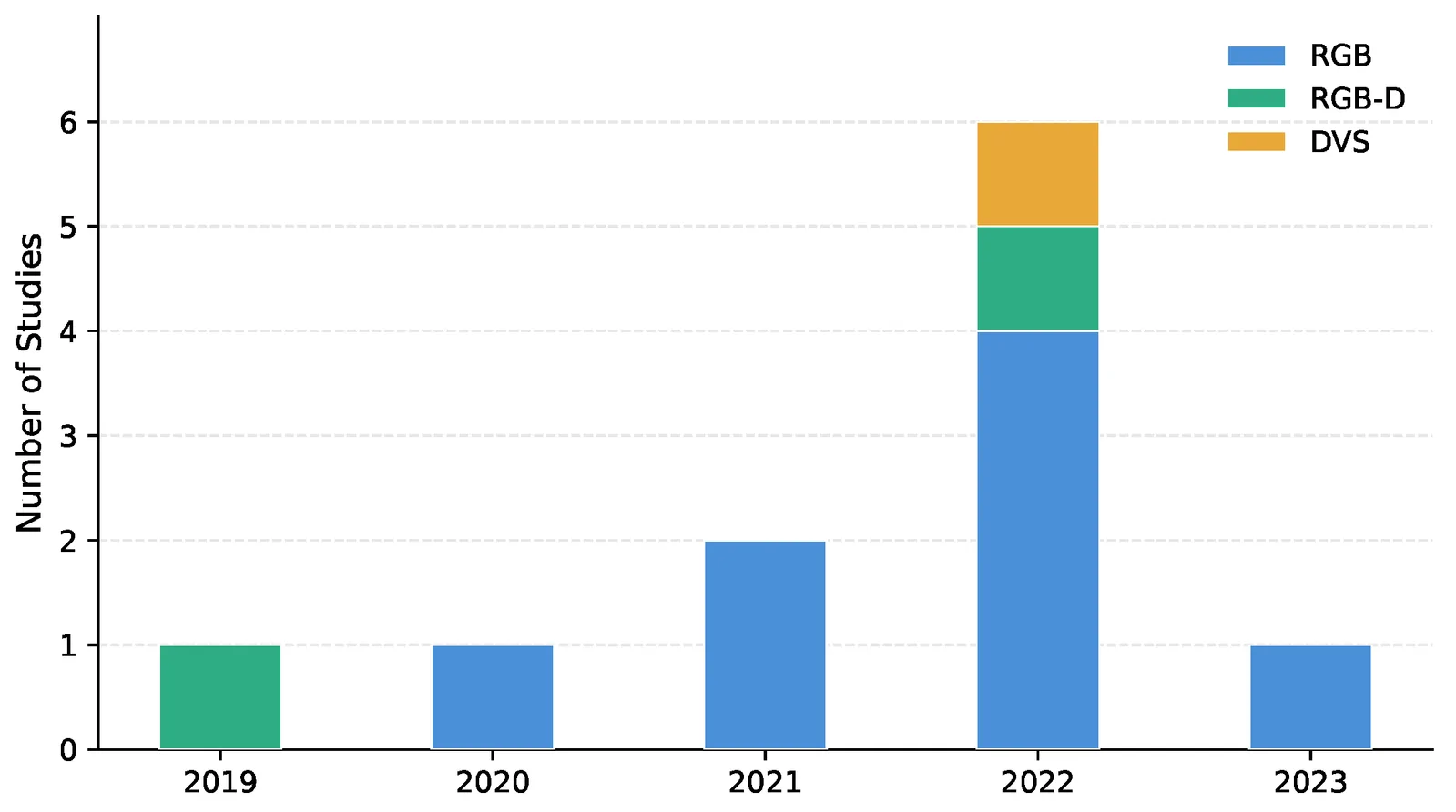
Year-wise distribution of input modalities across the 11 reviewed studies.

Figure 3 shows the year-wise distribution of input modalities across the chosen studies. These distributions reflect only the 11 reviewed studies and should be interpreted as descriptive characteristics of the included literature rather than general trends in the field. CNN classifiers perform end-to-end binary classification across these input types: skeleton-based features from Kinect depth data processed by a 1D-CNN [18], synthetic motion-encoded single frames from RGB [28], and event-based spatiotemporal cubes from DVS processed by a 3D-CNN [31].

Studies using object detectors treat fall detection as an object detection task with bounding boxes and class labels. The reported models are NanoDet-Lite [24], YOLO variants [25,33], and SSD-MobileNetV1 [32]. All these models are lightweight, featuring backbones such as MobileNetV1, ShuffleNet, or tiny architectures. This reflects the computational constraints of edge deployment, where real-time inference on devices with limited memory favors models with fewer parameters, even at the cost of reduced detection accuracy.

Hybrid approaches use CNNs for intermediate tasks rather than end-to-end classification. CNNs perform pose estimation or feature extraction, while secondary models such as LSTM or rule-based systems make the final decision. Rothmeier et al. [29] use MediaPipe Pose to extract skeletal keypoints and then compare machine learning and rule-based methods for classification. Menacho et al. [20] use optical flow to generate motion-based images, with CNN as the final classifier. Their system reported the lowest recall (41.47%) and precision (31.04%) among the reviewed studies. The authors attribute this lower performance to the use of a single optical-flow frame rather than a sequence of frames.

### 4.2. RQ2: Deployment Platforms

**Figure 4:**
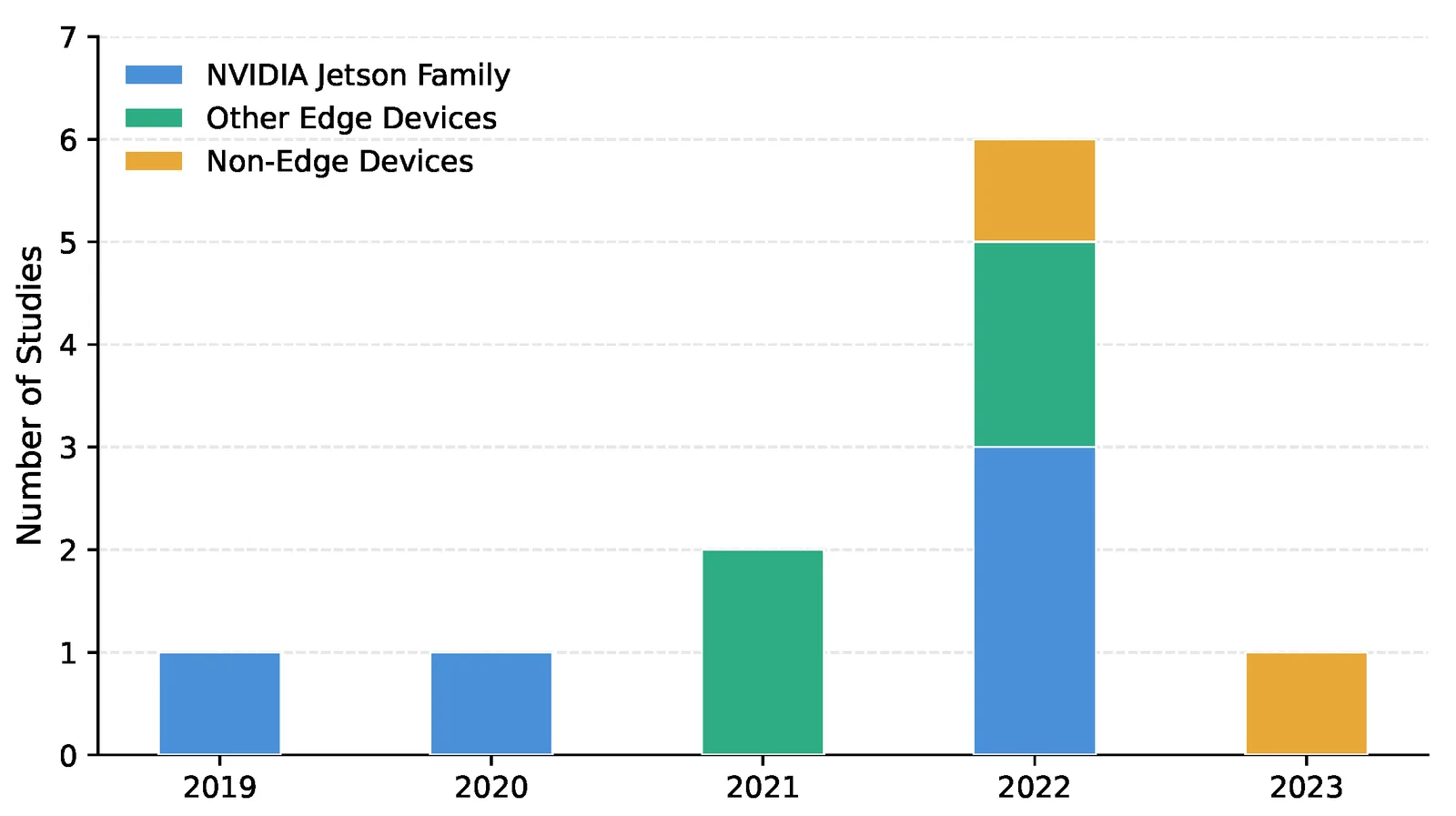
Year-wise distribution of deployment platforms across the 11 reviewed studies.

Among the analyzed studies, edge devices are the predominant hardware platform, with 9 out of 11 studies selecting this option. Within this group, 5 studies execute inference on NVIDIA Jetson boards, while 4 studies use CPU-only edge devices, including Raspberry Pi, LattePanda, and one ARM board. The remaining 2 studies deploy their systems on GPU-accelerated desktops. Figure 4 shows the year-wise distribution of deployment platforms across the chosen studies.

#### 4.2.1. Edge Devices

Table 4 summarizes the edge devices used in these studies. These devices include three NVIDIA Jetson models (AGX Xavier, TX2, and Nano), Raspberry Pi 4B, LattePanda V1, and one unspecified ARM board.

All Jetson boards are equipped with CPUs, but AI workloads are executed on the GPU, which is optimized for parallel computation. Only one study compares both options and reports a clear performance difference, achieving 23.16 fps on the GPU compared to 10.23 fps on the CPU [20]. In contrast, devices such as Raspberry Pi and LattePanda rely on CPU-only inference, which requires lightweight models and further optimization for real-time performance.

#### 4.2.2. GPU Servers

Both Hwang et al. [28] and Cheng et al. [33] deployed their models on the same desktop GPUs that were used for training. Hwang et al. [28] converted a PyTorch model to ONNX and increased the throughput from 162 to 204 fps on an RTX 3080. Cheng et al. [33] ran YOLOv5-Lite on an RTX 3060 (12 GB) at 640 × 480 resolution, achieving 3.12 ms per frame (approximately 320 fps). However, real-time camera processing reached only 25–33 fps due to system overhead. While these GPU-based setups deliver high inference speeds, they rely on high-power, non-portable hardware, making them impractical for deployment in home environments.

The reported inference speeds show that both GPU-assisted and CPU-only edge devices can meet the 10 fps threshold, but the results are not directly comparable across studies. Each study uses different models, input resolutions, datasets, and inference pipelines. Consequently, cross-device FPS normalization is not feasible within the available literature, and the reported FPS values should be interpreted as evidence of successful deployment rather than as directly comparable hardware benchmarks.

Desktop GPUs report the highest throughput, but their power use and physical footprint make them less practical for home deployment. Across all platforms, detection accuracy ranges from 84% to 99%. Given the substantial heterogeneity across datasets, models, devices, and evaluation protocols, no relationship between inference speed and accuracy can be established from the available studies. RQ3 examines the optimization strategies applied across these systems.

### 4.3. RQ3: Optimization Strategies and Trade-Offs

Achieving real-time inference on resource-constrained devices requires optimization beyond model selection. Across the reviewed studies, we identify three categories of optimization strategies: model pruning and compression, inference framework selection, and hardware-specific acceleration. Table 5 summarizes the strategies applied in each study.

**Model pruning and compression.** Several studies reduce model size to fit the computational limits of their target devices. Tsai et al. [18] applied pruning to reduce their network from 4.9 million to 35 thousand parameters while preserving accuracy on the Jetson TX2, achieving 15 fps. Prasad et al. [31] used constant sparsity pruning with a sparsity level of 0.7. They tested two pruned 3D-CNN variants: one using a frame-skip rate of 3 with 32 × 32 input frames, and another using the same frame-skip rate with 48 × 48 input frames. Pruning reduced computation by about 30 times for the 32 × 32 model and about 46 times for the 48 × 48 model. However, it also roughly doubled the memory footprint, likely because the pruned model stores additional sparse-weight information. Liu et al. [32] used Paddle Lite to compress their SSD-MobileNetV1 model for deployment on a Raspberry Pi 4B, reaching 14 fps.

**Inference framework selection.** The choice of inference framework can significantly affect throughput without modifying the model itself. Chen et al. [24] deployed their NanoDet-Lite model using ncnn, a high-performance inference library optimized for embedded CPUs, achieving 22.03 fps on the Raspberry Pi 4B. Hwang et al. [28] converted their PyTorch model to ONNX and increased throughput from 162 to 204 fps on an RTX 3080 desktop GPU. These cases demonstrate that inference-time gains are possible without retraining or modifying the model architecture.

**Hardware-specific acceleration.** Tohidypour et al. [27] applied the most extensive set of optimizations among the reviewed studies. They serialized their model using TensorRT with INT8 quantization, configured virtual memory and linear memory allocation to address the Jetson Nano’s 4 GB RAM limitation, and shifted video preprocessing to the GPU using a GStreamer pipeline. Before optimization, the model required 17 min to start and could not sustain real-time inference. After applying these combined strategies, startup dropped to under 2 min and inference reached 30 fps. Prasad et al. [31] additionally implemented frame skipping with a skip rate of 3 during deployment, reducing the number of frames processed per second to maintain real-time performance on LattePanda at 30 fps.

Together, these studies suggest that real-time inference on constrained devices may benefit from combining model optimization with runtime and system-level optimization. Yet these studies rarely compare detection performance before and after optimization. Only Tsai et al. [18] explicitly report that pruning preserved accuracy; for the other studies, we cannot determine whether optimization affected detection performance.

## 5. Discussion

### 5.1. Dataset Characteristics and Limitations

Table 6 summarizes the datasets used across the reviewed studies. All datasets with verifiable information were recorded in controlled settings, and none include older adults: all falls are simulated by young volunteers. This raises questions about how well these models generalize to older adults, whose fall dynamics differ in speed, posture, and protective responses [15].

NTU RGB+D is a general action recognition dataset in which falls appear as one class among sixty action classes. The remaining datasets are built for fall detection research, with activity sets that include falls and fall-alike activities such as sitting, lying, or crouching. One dataset could not be verified: the AIHub Airport dataset is cited as hosted on the Korean AI Hub platform, but the original hyperlink is broken. Furthermore, the AIHub data usage policy imposes several regulations and agreements with the Korea Intelligent Information Society Agency and the implementing agency for accessing and using their datasets, making it difficult to retrieve additional details about the dataset. We could not find associated documentation, so the subject count and fall-focus classification are marked as not available in the table.

A related limitation arises in the study by Prasad et al. [31], who do not collect native event-based recordings. Instead, they convert RGB video sequences into event streams by displaying videos on a monitor and capturing the resulting intensity changes with a DVS camera. This highlights the scarcity of large-scale DVS datasets for fall detection.

Fall events are rare by nature, which makes available datasets imbalanced and increases the risk of overfitting [18,31]. Menacho et al. [20] report that datasets cover only a few scenes and subjects, which may cause models to learn background patterns instead of fall dynamics. Chen et al. [24] note that real-world conditions such as partial occlusion or blurred body parts rarely appear in training data. Rothmeier et al. [29] confirm that models trained on benchmark datasets fail to generalize to unseen actions in real environments: the RNN model in their study could not handle common non-fall activities such as walking or standing up, because these activities were absent from the training data.

One limitation we encountered during our review was the lack of detailed information about the specific cameras used in the included studies. Most studies did not report camera specifications such as resolution, frame rate, or the presence of onboard processing capabilities. This information is important for understanding the deployability and computational requirements of real-time fall detection systems. Cameras with higher resolution and frame rates may improve detection accuracy but also increase the processing burden on the main computational unit. We recommend that future studies in this domain report detailed camera specifications and explore the use of cameras with onboard processing to optimize the balance between detection performance and computational efficiency.

Future work should prioritize datasets that include older adults performing both falls and daily activities in realistic home environments. Synthetic data generation and transfer learning from large-scale action recognition datasets offer complementary paths for expanding training data diversity without the cost and ethical constraints of recording real falls in vulnerable populations.

### 5.2. Evaluation Protocols

Evaluation protocols vary across the reviewed studies. Several authors train and test on a single dataset using unspecified split ratios [18,20,25]. Others combine datasets without defining the split strategy [22,24,28,29]. Several studies rely solely on custom datasets and omit evaluation details [27,32,33]. An exception is Prasad et al. [31], who define subject-based splits. No reviewed study performs cross-dataset evaluation. Shared benchmarks and evaluation protocols would improve reproducibility and enable fair comparison across studies.

### 5.3. Privacy and Alert Mechanisms

Four studies adopt input modalities or processing strategies that preserve privacy. Prasad et al. [31] use a dynamic vision sensor that captures only intensity changes rather than full images. Lu et al. [25] and Tsai et al. [18] use Kinect-based depth data, which reduces facial and texture information compared with RGB video. Tohidypour et al. [27] address privacy at the processing level: all inference runs on-device, and previously used frames are deleted from memory. Fall detection systems are often deployed in sensitive areas such as bedrooms and bathrooms, so future studies should evaluate privacy alongside technical performance.

Alert mechanisms are equally important for practical deployment. Only two studies integrate alert systems to inform caregivers [27,32]. Tohidypour et al. [27] state only that a notification is sent to a caregiver upon detection, without specifying the mechanism or timing. Liu et al. [32] describe sending an email notification after a fall is detected for 10 consecutive seconds. Neither study reports notification latency, severity grading, or alert reliability. Overall, alerting remains an underexplored component of vision-based fall detection systems, despite being critical to their practical effectiveness.

We identify three broad deployment profiles from the performance, power consumption, and indicative price data. We present these as general observations, not device recommendations. The prices in Table 4 reflect the French market in April 2026 and may not correspond to the costs at the time of the original studies. For low-cost home deployment, Raspberry Pi devices offer an accessible low-power option but require a CPU-optimized inference framework. For balanced performance and portability, GPU-equipped edge devices such as the NVIDIA Jetson family provide higher throughput at moderate power draw. Desktop GPUs achieve the highest throughput but their power draw and physical size make them less practical for home deployment.

### 5.4. Limitations of This Review

Deploying vision-based fall detection systems in real-world, resource-constrained environments remains difficult. Studies must show not only that their models are accurate, but also that they can run in real time under practical computational limits. Our inclusion criteria required each study to report inference speed on a specified device, which reduced the number of studies from 588 to 11. This criterion selected studies that had reached a real-time deployment stage, so the gaps we identify may reflect this subset rather than the whole field. In addition, no study from 2024 met all inclusion criteria, although our search covered 2019–2024. Another limitation of this review is that the search closed at the end of 2024 to allow sufficient time for screening, data extraction, and quality assessment; consequently, studies published in 2025 and early 2026 are not included in this study.

While directly comparing the accuracy of deployable and non-deployable systems is challenging due to differences in hardware, optimization, and evaluation settings, future studies should report both accuracy and runtime metrics across realistic deployment scenarios to better characterize the trade-offs between performance and deployability in real-world fall detection systems. These constraints limit how broadly we can generalize our conclusions. Still, they support a key finding: most studies do not report the runtime details needed to judge real-time deployment.

## 6. Conclusions

This review examined vision-based fall detection systems that report inference-time performance on physical hardware. From 588 records, only 11 met all inclusion criteria, confirming that deployment-validated systems remain rare in this field.

Three findings stand out. First, CNN-based architectures dominate algorithm choice, and the selected studies often use lightweight backbones to support edge deployment. Second, edge devices dominate deployment platforms, including NVIDIA Jetson boards, Raspberry Pi, LattePanda, and ARM-based hardware. Third, real-time inference on constrained devices depends on optimization strategies such as model pruning, quantization, inference framework selection, and hardware-specific acceleration. Yet only one study clearly reports whether optimization preserved detection accuracy, so its effect on detection performance remains unclear in most cases.

We also identified two broader limitations related to the datasets. The reviewed studies do not test their systems with older adults in real-world environments, and they rely mainly on small, controlled datasets with simulated falls. These limitations reduce confidence that current systems can generalize to practical elderly-care settings.

We encourage researchers to report deployment metrics such as inference latency and memory usage alongside detection accuracy. The field also needs benchmarks for cross-dataset evaluation, preferably with older adults in realistic environments. In addition, transfer learning from general action-recognition models may help when fall-specific data are limited. Distinguishing falls from visually similar actions such as sitting down or crouching remains a key challenge, particularly when relying solely on motion cues. Efficient vision-language models may improve scene understanding and help separate falls from similar actions, but their memory and compute requirements remain a challenge for resource-constrained devices. These steps would move vision-based fall detection from offline benchmarks toward practical elderly-care deployment.

## Figures and Tables

**Table 1:** Comparison of previous review articles on fall detection systems.

Article	Year	Systematic	Real-Time	Wearable	Scope	Vision
		Review	Performance		Ambient	
[10]	2020	✗	✓	✗	✗	✓
[9]	2020	✗	✗	✓	✓	✓
[2]	2021	✗	✗	✗	✗	✓
[11]	2022	✗	✗	✗	✗	✓
[12]	2024	✓	✗	✗	✗	✓
Ours	2026	✓	✓	✗	✗	✓

*Note:* ✓ = yes; ✗ = no.

**Table 2:** Logical structure of the search query used in the systematic review. The database-specific query strings are provided in the Supplementary Material.

(("Fall detection" OR "Fall accident") AND ("Video" OR "Deployment" OR "Camera" OR "Machine learning" OR "Deep learning" OR "CNN" OR "Neural network" OR "Vision based" OR "Real time" OR "Elderly")) AND NOT ("Radar" OR "Accelerometer" OR "Review" OR "Survey" OR "Gyroscope" OR "Wearable" OR "WiFi"))

**Table 3:** Overview of selected studies.

Ref.	Algorithm	Dataset	Input	Performance Metrics	Deployment	Inference (FPS)	Year
[18]	Depth skeleton extraction + 1D CNN	NTU RGB+D [19]	RGB-D	Acc-99.2% Rec-98.9% Prec-99.1%	Jetson TX2	15 FPS	2019
[20]	Optical Flow + Custom CNN	URFD [21]	RGB	Acc-88.55% Rec-41.47% Prec-31.04%	Jetson TX2	23.16 FPS (GPU) 10.23 FPS (CPU)	2020
[22]	Pose estimation (CNN) + LSTM	Multicam [23], URFD	RGB	**Multicam:** Rec-98.46% Spec-91.82% Acc-96.28% **URFD:** Rec-100.00% Spec-97.44% Acc-99.00%	ARM board	48 FPS	2021
[24]	CNN (NanoDet-Lite)	Custom dataset, URFD	RGB	mAP_50:95_-91.2% Rec-100.0% Prec-97.2%	Raspberry Pi 4	22.03 FPS (ncnn)	2021
[25]	CNN (YOLOv3-Tiny)	SDUFall [26]	RGB-D	Acc-98.7%	Jetson Xavier	20 FPS	2022
[27]	Custom CNN + LSTM (LRCN)	Custom dataset	RGB	Acc-84.44%	Jetson Nano	30 FPS	2022
[28]	CNN (EfficientNet-B0)	URFD, AIHub Airport [28]	RGB	**URFD:** Acc-97.14% Prec-100.00% Rec-93.33% Spec-100.00% **AIHub:** Acc-92.25% Prec-73.59% Rec-95.76% Spec-71.15%	GPU (RTX 3080)	162 FPS (PyTorch) 204 FPS (ONNX)	2022
[29]	MediaPipe Pose + RF/SVM/RNN/ Rule-based system	UP-Fall [30], URFD	RGB	Acc-94.40% Rec-90.90% Prec-96.60% F1-93.70%	Jetson Nano	15–18 FPS (deployed rule-based system)	2022
[31]	3D-CNN	DVS UR Fall (derived from URFD)	DVS	Acc-98.60% Rec-96.66% Spec-100.00%	LattePanda	30 FPS	2022
[32]	CNN (SSD-MobileNetV1)	Custom dataset	RGB	mAP_50_-92.70%	Raspberry Pi 4	14 FPS	2022
[33]	CNN (YOLOv5-Lite)	Custom dataset	RGB	Acc-92.10% Rec-85.60%	GPU (RTX 3060)	25–33 FPS	2023

*Note:* Abbreviations used in the Performance Metrics column: Acc—Accuracy; Prec—Precision; Rec—Recall; Spec—Specificity; F1—F1-score; mAP—mean Average Precision; FPS—Frames Per Second. Inputs: RGB—Red-Green-Blue image; RGB-D—RGB with Depth; DVS—Dynamic Vision Sensor (event camera).

**Table 4:** Comparison of edge devices reported in the studies of the systematic review.

Device	CPU	GPU	Power	AI Performance	Price	Approximate Cost Category
Jetson AGX Xavier	8× Carmel [34]	512-core Volta [34]	10–30 W [34]	32 TOPS [34]	32 GB-1302 € [35] 64 GB-1458 € [36]	High
Jetson TX2 ▲	2× Denver2,4× A57 [37]	256-core Pascal [37]	7.5–15 W [37]	1.33 TFLOPS [37]	8 GB-594 € [38]	Medium
Jetson Nano ▲	4× A57 [39]	128-core Maxwell [39]	5–10 W [39]	472 GFLOPS [39]	4 GB-210 € [40]	Low
LattePanda V1	4× Atom (x86-64) [41]	Intel HD Graphics 400 [41]	2 W [41]	N/A	2 GB-167 € [42] 4 GB-280 € [42]	Low
Raspberry Pi 4B	4× A72 [43]	VideoCore VI [43]	3.5–5 W [44]	N/A	4 GB-120 € [45] 8 GB-200 € [46]	Low

*Note:* Prices listed are indicative values corresponding to specific device variants available on the French market at the time of access (April 2026). Prices include value-added tax (VAT) and may vary depending on vendor and availability. The “Approximate Cost Category” column provides a more stable overview of the relative costs of the devices. ▲—Discontinued by NVIDIA at the time of writing.

**Table 5:** Optimization strategies applied in the reviewed studies.

Ref.	Device	Strategy	FPS	Trade-Off
[18]	Jetson TX2	Model pruning (4.9M to 35K params)	15	Accuracy preserved
[24]	Raspberry Pi 4	ncnn inference framework	22.03	Not reported
[27]	Jetson Nano	TensorRT (INT8) + virtual memory	30	Not reported
[28]	RTX 3080	PyTorch to ONNX conversion	204	None (26% speed gain)
[31]	LattePanda	Sparsity pruning (0.7), frame skipping (*k* = 3)	30	2× memory footprint
[32]	Raspberry Pi 4	Model compression via Paddle Lite	14	Not reported

**Table 6:** Characteristics of datasets used in the reviewed studies, listed in chronological order.

Dataset	Year	Format	Modality	Subjects	Elderly	Classes	Samples	Fall-Focused	Public
Multicam [23]	2010	Video	RGB	1	No	9	24	Yes	Yes
URFD [21]	2014	Video	RGB, Depth, IMU	5	No	2	70	Yes	Yes
SDUFall [26]	2014	Video	Depth	10	No	6	1800	Yes	Yes
NTU RGB+D [19]	2016	Video	RGB, Depth, IR, Skeleton	40	No	60	56,880	No	Yes
UP-Fall [30]	2019	Video	RGB, IMU, EEG, IR	17	No	11	561	Yes	Yes
AIHub Airport [28] ^†^	N/A	Video	RGB	N/A	No	2	10,000	N/A	No

^†^ Values reported by the citing study; the dataset could not be independently accessed (see Section 5).

## Data Availability

No new data were generated in this study. The study is based on published literature retrieved from academic databases detailed in the manuscript.

## Supplementary Materials

The following supporting information can be downloaded at https://www.mdpi.com/article/10.3390/s26165069/s1: Table S1: Item-level quality assessment scores for all 13 studies evaluated during the quality assessment stage; Document S1: Database-specific search queries used in the systematic review, including query strings, filtering steps, and record counts for each database.

## Abbreviations

The following abbreviations are used in this manuscript:

CNN	Convolutional Neural Network
LSTM	Long Short-Term Memory
DVS	Dynamic Vision Sensor
RGB-D	Red-Green-Blue with Depth
FPS	Frames per Second
mAP	mean Average Precision
ONNX	Open Neural Network Exchange
CPU	Central Processing Unit
GPU	Graphics Processing Unit
PRISMA	Preferred Reporting Items for Systematic Reviews and Meta-Analyses
