# Supplementary material for: Real-Time Vision-Based Fall Detection Systems for the Elderly: A Systematic Review
Source: Sensors (Basel). 2026 Aug 10;26(16):5069. doi: 10.3390/s26165069 (PMC13517638; doi:10.3390/s26165069)
Supplement: Supplementary file 1 [file sensors-26-05069-s001.zip › sensors-4404060-Document S1.pdf]

Supplementary Material: Database-Specific Search Queries

The following database-specific search strings were used for the systematic review. Searches were restricted to the title field and covered publications from 2019 to 2024. The final counts reported below correspond to records retained before duplicate removal.

1. PubMed

Search field: Title
Date filter: 2019/01/01–2024/12/31
Final count: 43 records

Query:

```
(
(
"Fall detection"[Title] OR "Fall accident"[Title]
)
AND
(
"Video"[Title] OR "Deployment"[Title] OR "Camera"[Title] OR
"Machine learning"[Title] OR "Deep learning"[Title] OR "CNN"[Title] OR
"Neural network"[Title] OR "Vision based"[Title] OR
"Real time"[Title] OR "Elderly"[Title]
)
)
NOT
(
"Radar"[Title] OR "Accelerometer"[Title] OR "Review"[Title] OR
"Survey"[Title] OR "Gyroscope"[Title] OR "Wearable"[Title] OR "WiFi"[Title]
)
AND 2019/01/01:2024/12/31[dp]
```

2. IEEE Xplore

Search field: Document Title
Date filter: 2019–2024
Additional filter: magazine articles excluded
Final count: 245 records

Query:

```
(
("Document Title":"Fall detection" OR "Document Title":"Fall accident")
AND
(
"Document Title":Video OR
"Document Title":Deployment OR
"Document Title":camera OR
"Document Title":"Machine learning" OR
"Document Title":"Deep learning" OR
"Document Title":CNN OR
"Document Title":"Neural network" OR
"Document Title":"vision based" OR
"Document Title":"real time" OR
"Document Title":elderly
)
)
AND NOT
(
"Document Title":radar OR
"Document Title":accelerometer OR
"Document Title":review OR
"Document Title":survey OR
"Document Title":gyroscope OR
"Document Title":wearable OR
"Document Title":WiFi
)
```

Filtering steps:
Initial search result: 523 records
After applying the 2019–2024 year filter: 247 records
After excluding 2 magazine articles: 245 records

3. ACM Digital Library

Search field: Title
Date filter: 2019–2024
Final count: 20 records

Query:

((Title:"Fall detection" OR Title:"Fall accident") AND (Title:Video OR Title:Deployment OR Title:camera OR Title:"Machine learning" OR Title:"Deep learning" OR Title:CNN OR Title:"Neural network" OR Title:"vision based" OR Title:"real time" OR Title:elderly)) AND NOT (Title:radar OR Title:accelerometer OR Title:review OR Title:survey OR Title:gyroscope OR Title:wearable OR Title:WiFi)

Filtering steps:
Initial search result: 40 records
After applying the 2019–2024 year filter: 20 records

4. Web of Science Core Collection

Search field: Title
Date filter: 2019–2024
Document types retained: Article, Meeting, Early Access
Language: English
Final count: 280 records

Query:

(
TI=("fall detection" OR "fall accident")
AND
TI=(video OR deployment OR camera OR "machine learning" OR "deep learning" OR CNN OR "neural network" OR "vision based" OR "real time" OR elderly)
)
NOT
TI=(radar OR accelerometer OR review OR survey OR gyroscope OR wearable OR WiFi)

Filtering steps:
After applying the 2019–2024 year filter: 487 records
After restricting the search to Web of Science Core Collection: 296 records
After retaining only Article, Meeting, and Early Access document types: 282 records
After excluding 2 non-English records: 280 records

Final records before duplicate removal:

PubMed: 43
IEEE Xplore: 245
ACM Digital Library: 20
Web of Science Core Collection: 280

Total: 588 records
